# Shift Work and the Relationship with Metabolic Syndrome in Chinese Aged Workers

**DOI:** 10.1371/journal.pone.0120632

**Published:** 2015-03-11

**Authors:** Yanjun Guo, Yi Rong, Xiji Huang, Hanpeng Lai, Xin Luo, Zhihong Zhang, Yuewei Liu, Meian He, Tangchun Wu, Weihong Chen

**Affiliations:** 1 Department of Occupational and Environmental Health, School of Public Health, Tongji Medical College, Huazhong University of Science and Technology, Wuhan, China; 2 Key Laboratory of Environment and Health in Ministry of Education & Ministry of Environmental Protection, and State Key Laboratory of Environmental Health (Incubating), School of Public Health, Tongji Medical College, Huazhong University of Science and Technology, Wuhan, China; Universität Bochum, GERMANY

## Abstract

**Background:**

Shift work is indicated to be associated with adverse metabolic disorders. However, potential effects of shift work on metabolic syndrome (MetS) and its components have not been well established.

**Methods:**

In total, 26,382 workers from Dongfeng-Tongji Cohort were included in this study. Information on shift work history was gathered through questionnaires and metabolic traits were measured. Logistic regression models were used to calculate the odds ratio (OR) and 95% confidence interval (CI) for long-term shift work related with MetS and each component, respectively. Further stratification analysis was performed to detect the differences on MetS between female and male shift workers.

**Results:**

Long-term shift work was associated with MetS without adjusting for any confounders. Compared with the group of non-shift work, the multivariate-adjusted ORs (95%CI) of MetS associated with 1–10, 11−20, and ≥20y of shift work were 1.05 (0.95−1.16), 1.14 (1.03−1.26), 1.16 (1.01−1.31), respectively. In female workers, we found a dose-response relationship that every 10 years increase in shift work was associated with a 10% (95% CI: 1%−20%) elevated OR of MetS, while no significant dose-response trend was found among male workers. Furthermore, shift work duration was significantly associated with ORs of high blood pressure (1.07, 1.01−1.13), long waist circumference (1.10, 1.01−1.20) and high glucose levels (1.09, 1.04−1.15). No significant association was observed between shift work and low HDL cholesterol) and raised triglyceride levels.

**Conclusions:**

Long-term shift work was associated with metabolic syndrome and the association might differ by gender in retired workers. Applicable intervention strategies are needed for prevention of metabolic disorders for shift workers.

## Introduction

Shift work is a prevalent employment practice in many industries [1]. Shift work refers to a wide range of work hour arrangements involving two or more teams (shifts). With the development of industry and economics, the demand of shift work is increasing all over the world. According to recent studies, there are more than 20% of all employees are shift workers in industrial countries [2,3], and the number of shift workers is still on the rise in China.

Shift work is recognized as a risk factor of many health outcomes by interrupting human circadian rhythm [4,5]. Circadian rhythm can have effect on sleeping and feeding patterns, and also in patterns of core body temperature, brain wave activity, hormone production and other biological activities[6]. Previous studies also confirmed a positive relationship between sleep deprivation and autonomic nervous system disorders. And autonomic nervous system disorders can cause chronic diseases in the long run [7,8]. All the above mechanisms contribute to the onset of chronic metabolic diseases when circadian rhythms changes.

Recently, accumulating evidences have shown that shift work is related with cardiovascular diseases [4,9] and type 2 diabetes [2,10] even in retired populations [11]. Some studies indicated that hypertension and type 2 diabetes were associated with metabolic syndrome (MetS) [12]. In particular, MetS induces an almost twice increased risk for coronary heart disease[13], a two to threefold increased risk for future ischemic stroke [14,15] and an even greater risk for diabetes [16]. Therefore, the studies on influence factors of MetS would be helpful for the prevention of the above diseases [12].

Several studies have been conducted to evaluate the association between shift work and metabolic syndrome (MetS), but the results are inconsistent. However, information on the duration of shift work was not available in most of these studies, and the sample sizes were relatively small [3,17,18]. In a recent meta-analysis, it was indicated that there was a significant positive dose–response relationship between night shift work duration and the risk of MetS [19]. However, few studies have established the effect of shift work duration on MetS after workers leaving shift positions. Furthermore, a meta-analysis have mentioned the gender differenes between shift work and MetS [19]and previous studies also have indicated a difference on the development of MetS between male and female [20], but no former study focused on the gender differenes between shift work and MetS.

Therefore, we conducted an evaluation between shift work and MetS on Dongfeng-Tongji cohort (DFTJ cohort) of 26,382 retired workers. Our objectives were to quantify the adverse effects of shift work and duration of shift work on MetS and realted indexes in retired workers. And to examined the gender differenes between shift work and MetS.

## Method

### Ethics Statement

The study protocol was approved by the institutional review boards of Dongfeng Motor Corporation (DMC) and Tongji Medical College Institutional review Board, School of Public Health, Tongji Medical College, Huazhong University of Science & Technology (Wuhan, Hubei, China). All participants provided written informed consent.

### Study Population

We conducted a cross-sectional study with a retrospective assessment of shift work history using the baseline data of DFTJ cohort which has reported in previous study [21]. In brief, 27,009 retired workers were included in the cohort who were covered by Dongfeng Motor Corporation’s (DMC’s) health care service systems and agreed to provide baseline blood samples and questionnaire information between September, 2008 and June, 2010. Standard questionnaires were used to collect information on demographics, occupational history, medications, self-reported medical history, and lifestyles. Physical examinations were performed at baseline to measure weight, height, waist circumference, blood pressure, blood lipids (total cholesterol, triglycerides, high-density lipoprotein cholesterol, and low-density lipoprotein cholesterol), fasting glucose, hepatic function, renal function, tumor biomarkers and some traits in urine. Information was collected by trained interviewers through face to face interviews. Trained investigators entered questionnaires into computer twice using EpiData software. And a group of trained investigators performed quality control by rechecking the entered data. In this study, participants were excluded if they provided no information on demographics (*n* = 204), occupational history (*n* = 241), sleeping quality (*n* = 101) or shift work duration (*n* = 81). Finally, 26,382 participants were included.

### Ascertainment of Shift Work

Information on shift work history was collected via questionnaires. Shift work was defined as any work schedule involving unusual or irregular working hours as opposed to a normal daytime work schedule: 8:00AM to 17:00PM in this study. There are 3 kinds of shift work in Dong Feng Motor Company: two shifts (12 hours a shift); three shifts (8 hours a shift) and four shifts (6 hours a shift). The workers in any kind of shift work have to work at night. And participants with a history of shift work for at least one year were recognized as shift workers. According to the distribution of shift work duration, we categorized shift work into four categories: never, 1–10, 11–19, and ≥ 20 years for further analysis.

### Ascertainment of Metabolic Syndrome

After an overnight fast, all participants got physical examinations by trained physicians, nurses and technicians at health examination centre of Dongfeng Central Hospital. The fasting glucose, blood lipids were tested by ARCHITECT CI8200, Abbott,USA. In this study, we defined MetS according to the diagnostic criteria proposed by the Adult Treatment Program III of the National Cholesterol Education Program (NCEP ATP III, 2005), participants were recognized as MetS patients if they met three or more of the following variables and cutoff points: (1) Fasting triglyceride≥1.69 mmol/L (150 mg/dL); (2) HDL cholesterol: Men<1.04 mmol/L (40 mg/dL), Women<1.29 mmol/L (50 mg/dL); (3) Fasting glucose: ≥5.5 mmol/L (100 mg/dL); (4) Waist circumference: men ≥90 cm, women ≥80 cm (modified for the recommended cut-off for the Asia-Pacific region); (5) Systolic blood pressure ≥130 mmHg and/or diastolic blood pressure≥85 mmHg.

### Statistical Analyses

Demographic, lifestyle and occupational characteristics distributions at baseline were compared between shift workers and day workers by Chi-square test and Student-T test for classified variables and continuous variables respectively. Logistic regression models were used to calculate the odds ratio (OR) and 95% confidence interval (CI) for MetS and each component, respectively, according to shift work and duration of shift work. Linear regression models were used to estimate the ORs per 10 years increase of shift work.

For further analysis, we conducted stratification analysis to detect the gender differences on MetS of shift workers. Then the odds ratios of every group were calculated using the following logistic regression models.

In model1, we did not control any of the confounders. In model2, we only adjusted for gender and age (continuous). In model3, we further adjusted for body mass index (BMI) (continuous) except the confounders in model2. And in the multivariate-adjusted models, we adjusted for gender, age (continuous), race (Han, others), marital status (single or divorced, married), education (junior high school or below, senior high school or above), current smoking status (no, yes), passive smoking (no, yes), current drinking status (no, yes), tea or coffee consumption (no, yes), life stress (no, yes), physical activity (no, yes), retirement duration (continuous) and body mass index (BMI) (continuous). In all the models, the comparison group of this study was day workers.

In this study, physical activities contain many aspects of activities, such as, climbing, walking, dancing, cycling, running, swimming and so on. Physical activity is defined as “yes” if the participant exercises ≥ 2 times per week and each time ≥ 20 minutes. Life stress (no, yes) was self-reported, and life stress is defined as ‘yes’ if the participants feel nervous, upset or even despair of daily life equal or more than 3 times per week. BMI was included in the multivariate-adjusted models, because previous studies have suggested that BMI was a mediate variable for diabetes [22]. All *p*-values were two sided with a significant level at 0.05, and data were analyzed with SAS 9.1 (SAS Institute Inc. Cary, NC, USA).

## Results

A total of 26,382 participants (11,783 male workers and 14,599 female workers) were included in this study. The average age of the whole population was 63.6 years old in 2008. Distribution of baseline characteristics and occupational history were presented in Table 1. A total of 9,088 participants were shift workers were engaged in shift work for more than 1 year, among which 76.2% shift workers were mainly engaged in two-shifts, 22.6% were three-shifts and1.2% were four-shifts. There are some differences in age distributions of day workers and shift workers. About 39.34% shift workers are under 60 years old while only 30.7% of day workers are less than 60 years old. Female shift workers were more than male workers in this motor factory, and the difference is statistically significant. Shift workers (69%) had a higher percentage of less education when compared with day workers (62%). Smoking and drinking were significantly higher in shift workers than day workers (*p*<0.01). There was no significantly difference in daily exercise between shift workers and day workers

**Table 1:** The characteristics of the study population according to shift work.

Variables	Total	Shift Work		*P* value
		Never	≥1 Year	
**Gender**				0.008
Male	11,783	7,622(44.07)	4,161(45.79)	
Female	14,599	9,672(55.93)	4,927(54.21)	
**Age**				<0.0001
≤55	4,054	2,364(13.67)	1,690(18.60)	
56–60	4,831	2,946(17.03)	1,885(20.74)	
61–65	7,673	4,935(28.54)	2,738(30.13)	
66–70	4,603	3,302(19.09)	1,301(14.32)	
≥71	5,221	3,747(21.67)	1,474(16.21)	
**BMI**				0.207
<18.5	1,368	905(5.23)	463(5.09)	
18.5–24	10,934	7,102(41.07)	3,832(42.17)	
24–28	10,384	6,818(39.42)	3,566(39.24)	
≥28	3,696	2,469(14.28)	1,227(13.50)	
**Education**				<0.0001
junior high school or below	17,264	10,913(63.10)	6,351(69.88)	
senior high school or above	9,118	6,381(36.90)	2,737(30.12)	
**Race**				0.156
Han	25,991	17,048(98.58)	8,943(98.40)	
Others	391	246(1.42)	145(1.60)	
**Marriage**				0.037
Married	22,968	15,002(86.75)	7,966(87.65)	
Unmarried or Divorced	3,414	2,292(13.25)	1,122(12.35)	
**Retirement duration**				<0.0001
<10	6,747	4,151(24.00)	2,596(28.57)	
10–15	5,625	3,481(20.13)	2,144(23.59)	
15–20	9,041	6,123(35.41)	2,918(32.11)	
≥20	4,969	3,539(20.46)	1,430(15.73)	
**Current Smoking**				
No	21,534	14,400(83.27)	7,296(79.64)	<0.0001
Yes	4,686	2,894(16.73)	1,792(20.36)	
**Current Drinking**				<0.0001
No	20,887	13,879(80.25)	7,019(77.20)	
Yes	5,484	3,415(19.75)	2,069(22.80)	
**Exercise**				0.121
No	3,003	2,018(11.67)	985(10.84)	
Yes	23,379	15,276(88.33)	8,103(89.16)	

Abbreviation: BMI, body mass index. Variable are given as frequency for categorical data. *P* value was calculated by Chi-square test for categorical data

Table 2 shows the differences of waist circumference, blood pressure, fasting glucose, lipids, and blood haematological traits between shift workers and day workers. Shift workers had higher blood pressure either in systolic blood pressure (SBP) or in diastolic blood pressure (DBP) (*p*<0.01). Compared with day workers, fasting glucose was higher in shift workers (*p*<0.01). High-density lipoprotein (*p*<0.01) was lower in shift group, while low-density lipoprotein (*p* = 0.408), total cholesterol (*p*<0.01), triglyceride (*p* = 0.804) were higher than day workers.

**Table 2:** The baseline levels of some medical examination traits of the participants according to shift work.

Variables(means±sd)	Total	Shift Work		*P* value
		Never	≥1 Year	
**Weight**	63.50±10.44	63.49±10.44	63.53±10.44	0.757
**Waist**	83.24±9.51	83.32±9.48	83.09±9.57	0.073
**SBP**	129.94±18.74	129.46±18.76	130.20±18.72	0.003
**DBP**	77.88±10.89	77.58±10.86	78.47±10.94	<0.0001
**Haematological traits**				
RBC (t/l)	4.57±0.47	4.57±0.48	4.56±0.46	0.187
WBC (g/l)	6.07±1.68	6.06±1.68	6.08±1.68	0.298
Haemoglobin (g/l)	136.57±14.42	136.15±14.21	137.38±14.78	<0.0001
Platelet count (g/l)	186.92±56.86	184.96±55.67	190.60±58.87	<0.0001
**Fasting glucose (mmol/l)**	6.07±1.74	6.01±1.73	6.10±1.75	<0.0001
**Lipids**				
TC (mmol/l)	5.18±0.98	5.19±0.98	5.16±0.98	0.024
TG (mmol/l)	1.46±1.15	1.46±1.17	1.46±1.11	0.896
HDL-C (mmol/l)	1.44±0.41	1.44±0.40	1.43±0.43	0.029
LDL-C (mmol/l)	3.02±0.84	3.02±0.84	3.02±0.83	0.776

Abbreviation: SBP, Systolic blood pressure; DBP, diastolic blood pressure; RBC, red blood cell; WBC, white blood cell; TC, total cholesterol; TG, total triglycerides; HDL-C, high-density lipoprotein cholesterol; LDL-C, low-density lipoprotein cholesterol.

*P* values were calculated by independent sample t-test for numerical data.

We studied the relationship between shift work, shift work duration and MetS (Table 3). For the whole population, shift work was strongly associated with MetS without adjusting any potential confounders (OR = 1.17, *p*<0.0001). But, the association became weakened after adjusting gender and age (OR = 1.05, *p*>0.05). We observed that the ORs (95%CI) for female and male workers were 1.12 (1.04–1.21) and 1.13 (1.01–1.26) respectively. Compared with day workers, the ORs for participants with 1–10, 11–20, and ≥20 y of shift work were 1.05 (0.95–1.16), 1.14 (1.03–1.26), 1.16 (1.01–1.31) in model 4, respectively. In the secondary analysis, we examined the effect of shift work in male and female workers. The ORs for participants with 1–10, 11–20, and ≥20 y of shift work were 1.15 (0.94–1.41), 1.26 (1.02–1.50), and 1.09 (1.02–1.16) in male workers, as well as 1.02 (0.91–1.15), 1.13 (1.00–1.28), and 1.22 (1.11–1.33) in female workers, respectively. And in female workers, we found that every 10 years increase in shift work was associated with a 10% (95% CI: 1%-20%) elevated ORs of MetS. No significant shift work duration dependent association was found in male workers although ORs of MetS (OR = 1.15; 95%CI: 1.01–1.30) was significantly increased.

**Table 3:** Odds ratio of metabolic syndrome according to shift work duration.

	Duration of Shift Work (Years)				ORs per 10 y increase of shift work	*P* value for Trend
	Never	≤10	11–20	≥20		
**Total (OR:95%CI)**						
Model 1	1.00(referent)	1.02(0.93–1.11)	1.05(0.96–1.16)	1.36(1.22–1.51)	1.17(1.10–1.24)	<0.001
Model 2	1.00(referent)	1.04(0.95–1.14)	1.08(0.98–1.19)	1.34(1.21–1.49)	1.05(1.01–1.09)	<0.001
Model 3	1.00(referent)	1.08(0.98–1.19)	1.10(0.99–1.21)	1.36(1.22–1.52)	1.02(1.01–1.03)	<0.001
Model 4	1.00(referent)	1.05(0.95–1.16)	1.14(1.03–1.26)	1.16(1.01–1.31)	1.01(0.98–1.04)	0.072
**Male**						
Model 1	1.00(referent)	1.12(0.92–1.36)	1.21(1.01–1.41)	1.10(0.92–1.30)	1.07(0.96–1.18)	0.058
Model 2	1.00(referent)	1.12(0.92–1.36)	1.25(1.01–1.48)	1.11(0.98–1.24)	1.07(0.95–1.19)	0.064
Model 3	1.00(referent)	1.15(0.94–1.40)	1.23(1.02–1.44)	1.09(1.01–1.17)	1.06(0.94–1.18)	0.072
Model 4	1.00(referent)	1.15(0.94–1.41)	1.26(1.02–1.50)	1.09(1.02–1.16)	1.07(0.96–1.18)	0.068
**Female**						
Model 1	1.00(referent)	1.00(0.89–1.11)	1.13(1.02–1.25)	1.30(1.13–1.47)	1.13(1.04–1.23)	<0.001
Model 2	1.00(referent)	1.02(0.92–1.14)	1.11(0.99–1.24)	1.20(1.04–1.36)	1.11(1.01–1.21)	<0.001
Model 3	1.00(referent)	1.02(0.91–1.14)	1.13(1.00–1.28)	1.21(1.08–1.34)	1.10(1.01–1.21)	<0.001
Model 4	1.00(referent)	1.02(0.91–1.15)	1.13(1.00–1.28)	1.22(1.11–1.33)	1.10(1.01–1.20)	<0.001

Model 1: single factor logistic regression.

Model 2: adjusted for age (continuous), gender (male. female).

Model 3: adjusted for age (continuous), gender (male. female), body mass index (BMI) (continuous).

Model 4: adjusted for age (continuous), gender (male. female), body mass index (BMI) (continuous), race (Han, others), marital status (single or divorced, married), education (junior high school or below, senior high school or above), current smoking status (no, yes), passive smoking (no, yes), current drinking status (no, yes), tea or coffee consumption (no, yes), life stress (no, yes), physical activity (no, yes), and retirement duration (continuous).The reference group was day workers.

In Table 4, we observed the relationship between each component of MetS, shift work and shift work duration. The level of glucose, waist circumference, and blood pressure increased with the extension of shift work duration. We discovered that every 10 years increase in shift work years were associated with 5% (1%-9%), 13% (4%-22%), and 6% (3%-9%) elevated ORs for glucose, waist circumference, and blood pressure, respectively. Triglyceride significantly increased in 11–20 years shift work group after adjusted for potential confounders (OR = 1.11, *p*<0.0001). HDL-cholesterol decreased mainly in 1–10, and 11–20 years group, the ORs (95%CI) were 1.15 (1.04–1.27), 1.14 (1.02–1.26), respectively.

**Table 4:** ORs of each component of metabolic syndrome according to duration of shift work.

Component(OR:95%CI)	Duration of Shift Work(Years)				ORs per 10 y increase of shift work	*P* value for Trend
	Never	≤10	11–20	≥20		
**Triglyceride**						
Model 1	1.00(referent)	1.00(0.92–1.09)	1.08(0.98–1.18)	1.04(0.95–1.15)	1.01(0.96–1.06)	0.088
Model 2	1.00(referent)	0.99(0.90–1.08)	1.07(0.98–1.18)	1.01(0.92–1.11)	1.03(0.97–1.09)	0.096
Model 3	1.00(referent)	1.03(0.94–1.12)	1.11(1.01–1.22)	1.02(0.92–1.12)	1.03(0.97–1.09)	0.079
Model 4	1.00(referent)	1.03(0.94–1.12)	1.11(1.01–1.21)	1.01(0.92–1.12)	1.03(0.97–1.09)	0.085
**HDL Cholesterol**						
Model 1	1.00(referent)	1.28(1.18–1.40)	1.11(1.01–1.23)	1.48(1.33–1.65)	1.21(1.12–1.31)	<0.001
Model 2	1.00(referent)	1.14(1.03–1.25)	1.12(1.01–1.23)	1.04(0.92–1.18)	1.04(0.96–1.12)	0.064
Model 3	1.00(referent)	1.15(1.04–1.27)	1.14(1.02–1.26)	1.03(0.91–1.17)	1.04(0.96–1.12)	0.079
Model 4	1.00(referent)	1.15(1.04–1.27)	1.14(1.02–1.26)	1.03(0.91–1.17)	1.04(0.96–1.12)	0.072
**Glucose**						
Model 1	1.00(referent)	1.07(1.01–1.13)	1.14(1.05–1.23)	1.25(1.16–1.35)	1.06(1.01–1.11)	<0.001
Model 2	1.00(referent)	1.11(1.03–1.21)	1.14(1.05–1.23)	1.23(1.14–1.33)	1.07(1.02–1.12)	<0.001
Model 3	1.00(referent)	1.07(1.02–1.12)	1.07(1.01–1.13)	1.16(1.07–1.25)	1.05(1.01–1.09)	<0.001
Model 4	1.00(referent)	1.06(1.02–1.10)	1.06(1.01–1.11)	1.15(1.07–1.24)	1.05(1.01–1.09)	<0.001
**Waist Circumference**						
Model 1	1.00(referent)	1.01(0.89–1.14)	1.13(1.01–1.26)	1.70(1.47–1.96)	1.34(1.23–1.45)	<0.001
Model 2	1.00(referent)	1.03(0.91–1.17)	1.01(0.90–1.13)	1.26(1.08–1.47)	1.12(1.01–1.23)	<0.001
Model 3	1.00(referent)	1.07(0.94–1.22)	1.24(1.08–1.43)	1.23(1.10–1.36)	1.13(1.03–1.23)	<0.001
Model 4	1.00(referent)	1.07(0.94–1.21)	1.25(1.09–1.44)	1.25(1.15–1.35)	1.13(1.04–1.22)	<0.001
**Blood Pressure**						
Model 1	1.00(referent)	1.00(0.92–1.08)	1.07(1.00–1.15)	1.01(0.93–1.09)	1.01(0.96–1.06)	0.052
Model 2	1.00(referent)	1.04(0.96–1.13)	1.05(0.98–1.14)	1.01(0.93–1.09)	1.04(0.99–1.09)	0.059
Model 3	1.00(referent)	1.03(0.95–1.12)	1.06(0.98–1.14)	1.11(1.02–1.20)	1.06(1.02–1.10)	<0.001
Model 4	1.00(referent)	1.04(0.95–1.13)	1.06(0.98–1.15)	1.12(1.03–1.21)	1.06(1.03–1.09)	<0.001

Model 1: single factor logistic regression.

Model 2: adjusted for age (continuous), gender (male. female).

Model 3: adjusted for age (continuous), gender (male. female), body mass index (BMI) (continuous).

Model 4: adjusted for age (continuous), gender (male. female), body mass index (BMI) (continuous), race (Han, others), marital status (single or divorced, married), education (junior high school or below, senior high school or above), current smoking status (no, yes), passive smoking (no, yes), current drinking status (no, yes), tea or coffee consumption (no, yes), life stress (no, yes), physical activity (no, yes), and retirement duration (continuous).The reference group was day workers.

## Discussion

Our findings showed elevated ORs for MetS in both male and female workers after 10 years of shift work and demonstrated a gender difference on the relationship between shift work and MetS. The putative relation between shift work and MetS was investigated in a number of studies [18,23,24]. But, no previous study observed the long-term effect on MetS and its components according to shift work duration.

Three prospective studies revealed that shift work was an independent risk factor for MetS [3,12,25]. Unfortunately, these studies did not evaluate the risk according to shift work duration. Our studies suggested that the ORs for MetS increased with the extension of shift work duration. Every 10 years increase of shift work was related to 17% increase of OR in the unadjusted model. The increased ORs of MetS associated with shift work duration is consistent with previously reported positive relation of shift work with type 2 diabetes [2] and cardiovascular diseases [4,26].

There are some potential mechanisms underlying this relation. First, shift work was confirmed to disturbing the regular circadian rhythms. And a wide range of biological processes are regulated by the circadian rhythms, including sleep-wake cycles, body temperature, energy metabolism, cell cycle, and hormone secretion [2,6].Workers with long duration of shift work were suffering from chronic misalignment between the endogenous circadian timing system and the behavior cycles. The prevalent chronic misalignment in shift workers might result in a decrease in leptin, increase in glucose and insulin, increase in mean arterial blood pressure, and reduced sleep efficiency [27]. Second, shift work caused sleep deprivation, changes in sleep patterns, and reducing secretion of melatonin. Monk and our previos studies confirmed that shift workers suffered from worse sleep comparing with day workers, even after retirement [11,28]. Shift workers were more likely to sleep less at night and have worse sleeping quality. Accumulating studies proved that many adverse health effects such as anxiety, endocrine disorders could hit the body because of sleep deprivation [29–31]. Third, factors such as light at night, noises in the daytime could cause some unfavorable changes to blood pressure, glucose, endocrine, lipids, and cardiac activity. These changes also contribute to the adverse effect of shift work on MetS [31–33]. Fourth, health risk behaviors (mainly smoking and irregular meals) related to shift work is another potential reason for the relationship of shift work and MetS. Former studies have showed that shift workers were more likely to be current smokers [2]. The percentage of current smoking for shift workers was also higher than day workers in this study. Irregular meals may cause unfavorable changes in glucose, BMI and insulin, although studies showed small differences in nutritional intake or eating patterns between shift workers and day workers [34].

Previous studies have discovered the differences in the development of MetS between male and female [20,35,36]. Xu and his partners found that Chinese males got higher prevalence of MetS and its components, more complex and risky combinations of abnormal components, and faster development of MetS [35]. Our findings suggested that shift work strongly related to MetS in unadjusted model and that relation was weakened after adjusted for gender and age. In the following analysis, we further observed the differences in two subgroups: male and female. Compared with day workers, male shift workers were significantly higher in ORs of MetS after adjusting for multiple potential confounders than female shift workers. Recently, a meta-analysis has noticed the heterogeneity of female and male shift workers [19]. And there are several reasons for the difference between male and female shift workers[37]. Firstly, it is confirmed that there are differences in vitamin D insufficiency between female and male shift workers in published evidences. It was assumed that night work may decrease the sunlight exposure and subsequently reduce vitamin D levels while male workers worked longer as shift workers than female workers in many industries [38]. And, National Health and Nutrition Examination Surveys suggested that vitamin D intake and higher circulating vitamin D levels were associated with lower prevalence of MetS [39]. Secondly, male workers were more likely to have more risk health behaviors, such as higher fat diet, more food consume at night and longer sleeping duration. These behaviors were confirmed to elevate risk for metabolic disorders, cardiovascular diseases and diabetes [40,41]. Thirdly, male workers have a more activated sympathetic nervous system, greater endothelial dysfunction, and different renal sodium handling. These factors increased the susceptibility of MetS [42–44].

Furthermore, we not only established the association between shift work and MetS, but also the connection with every component of MetS according to shift work duration. We observed increased ORs in elevated blood pressure, waist circumference and glucose levels with the extension of shift work, but we did not examine significantly elevated ORs in low HDL cholesterol and raised triglyceride. This was partly consistent with the outcome of several prospective studies [3,45]. So far, this was the first study to prove the association of shift work duration and every component of MetS. This will be a significant important finding for prevention of metabolic related disorders among shift workers because of an increasing population on shift work worldwide.

The strengths of this study include its large sample size and detailed information on duration of shift work and other confounders. So far as we know, this is the largest study of retired workers to study shift work and its association with MetS and its components. We still have several limitations. First, we did not collect data on dietary patterns, but studies have confirmed that there are only very small differences in nutritional intake or eating patterns between shift workers and day workers. Second, we did not have information on income, occupational hazards and detailed environmental exposure; therefore we were unable to evaluate the confounding influences of occupational hazards and environmental exposure. However, the participants are all from one big company and living in one town and the environment or incomes were likely to be relatively homogenous.
